# High-Resolution Intact Protein Analysis via Phase-Modulated, Stepwise Frequency Scan Ion Trap Mass Spectrometry

**DOI:** 10.1021/acs.analchem.4c02775

**Published:** 2024-09-06

**Authors:** Fang-Hsu Chen, Chun-Yen Cheng, Szu-Wei Chou, Cheng-Han Yang, I-Chung Lu, Ming-Long Yeh

**Affiliations:** † Department of Biomedical Engineering, National Cheng Kung University, Tainan City 701, Taiwan; ‡ AcroMass Technologies Inc., Hukou, Hsinchu 30352, Taiwan; § Department of Chemistry, National Chung Hsing University, Taichung City 40227, Taiwan; ∥ Medical Device Innovation Center, National Cheng Kung University, Tainan City, 701, Taiwan

## Abstract

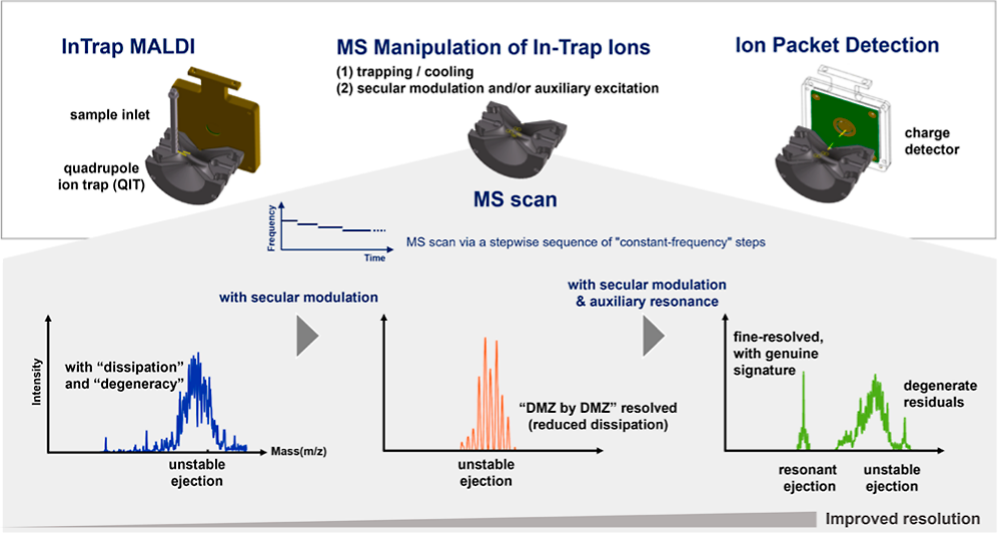

Mass spectrometry (MS) using an electron multiplier for intact protein analysis remains limited. Because of the massive size and complex structure of proteins, the slow flight speed of their ions results in few secondary electrons and thus low detection sensitivity and poor spectral resolution. Thus, we present a compact ion trap-mass spectrometry approach to directly detect ion packets and obtain the high-resolution molecular signature of proteins. The disturbances causing deviations of ion motion and mass conversion have been clarified in advance. The radio frequency waveform used to manipulate ions is proposed to be a sequence of constant-frequency steps, interconnected by short time-outs, resulting in least dispersive distortion. Furthermore, more such constant-phase conjunctions are arranged in each step to compensate for fluctuations resulting from defects in the system and operation. In addition, two auxiliary pulses are generated in the right phase of each step to select ions of a specific secular state to detect one clean and sharp spectral line.This study demonstrates a top-down approach for the MS measurement of cytochrome C molecules, resulting in a spectral profile of the protein in its natural state at a resolution of 20 Da. Additionally, quick MS scans of other proteins were performed.

## Introduction

Mass spectrometry (MS) can resolve molecular ions by their mass-to-charge ratio (*m*/*z*). MS’s rapid detection capability meets most requirements for protein identification in proteomic research and clinical diagnosis.^1,2^ However, the top-down MS approach shows drawbacks when analyzing massive and complex proteins.^3^ Thus, peptide hydrolysis for protein fragmentation, followed by using a database, is the common approach for quickly identifying proteins through their molecular fragments. Advanced MS instruments using time-of-flight (ToF), orbitrap, or cyclotron resonance can attain higher resolution at the expense of volume vacuum, making them costly.^4−6^ In addition, the quadrupole ion trap (QIT), also known as the Paul trap, was developed as a miniature MS system because of its compact and highly adaptable configuration to manipulate ions.

**Figure 1:**
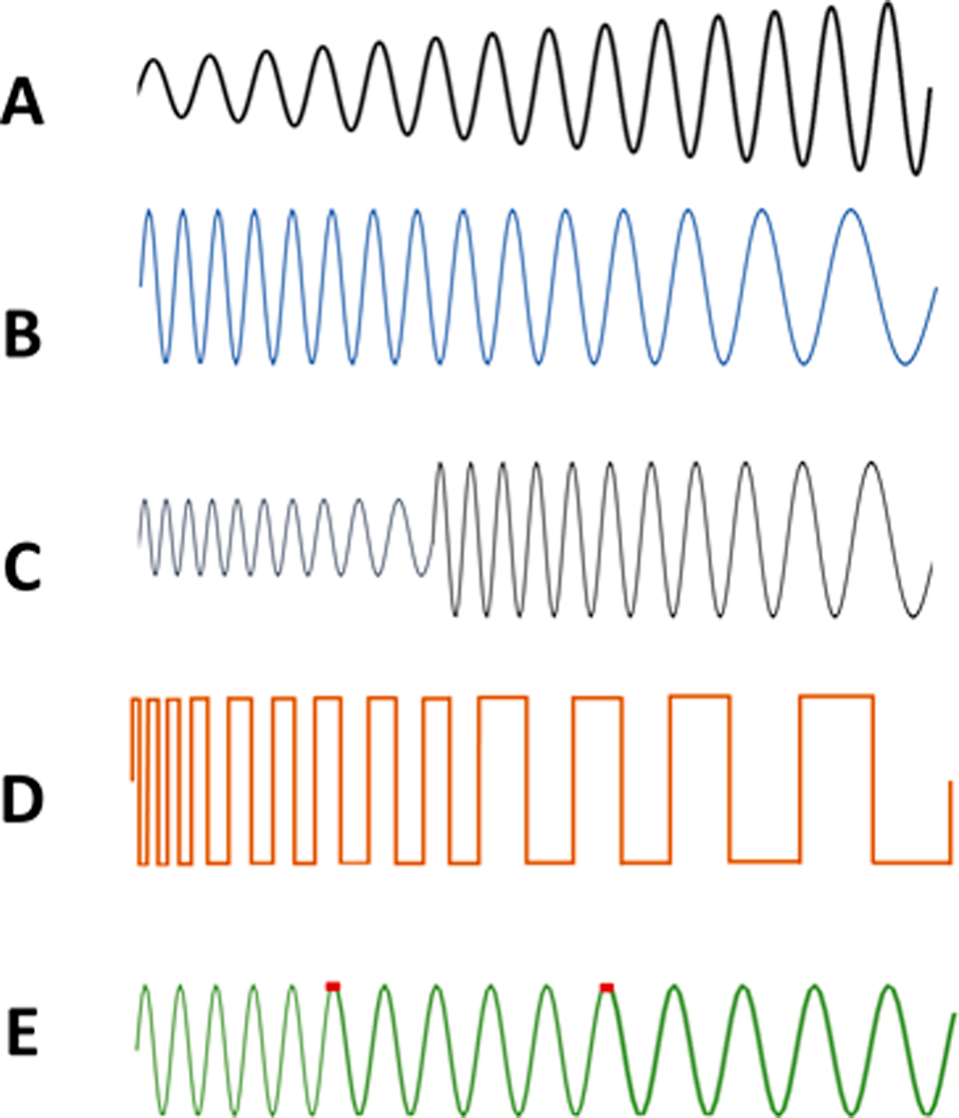
Schematic of different RF waveforms for ion trap-mass spectrometry. (A) MS scan by continuously increasing amplitude, with a fixed frequency (amplitude scan).^10,11^ (B) MS scan by continuously decreasing the frequency, with a fixed amplitude (frequency scan).^15^ (C) Hybrid frequency scan mode.^16^ (D) MS scan via a continuous frequency-changing square wave like DIT MS.^13,14^ (E) MS scan via a stepwise frequency-changing sequence of constant-frequency steps, wherein a short time-out in the RF waveform is used to connect the steps of different frequencies. The red spot indicates the location of such time-out.^17^ All these MS scans are arranged according to their stability diagram, derived from the dynamic equation of in-trap motion for closed orbits.

The Paul trap, composed of one ring electrode and two end-cap electrodes, is mainly radio frequency (RF) voltage-driven to trap ions inside the hyperboloid potential.^7−9^ In the case of small molecules, one linear scan in the waveform amplitude allows charged particles to be ejected and detected according to their nominal *m*/*z* values.^10,11^ Many other “frequency scan” waveforms have been devised to relocate or extend the mass range (Figure 1). Nevertheless, spectral broadening and shifting are of concern because of the switching RF waveform timing.^12^ In particular, the square waveform of digital ion trap (DIT) spectrometry provides a subtle integration of driving and switching (Figure 1D).^13,14^

The oscillatory motion (Figure S3) of in-trap ions generally exhibits little synchronization because every ion’s trajectory is continuously disrupted by buffer gas molecules and many other in-trap ions, not merely by nonideal electric confinement such as RF waveform defects (Supporting Information Section 1).^8,18^ These in-trap fluctuations are worsened for massive and slow protein ions, degrading the spectral resolution and detection efficiency of conventional QIT spectrometry that uses an electron multiplier to detect secondary electrons, resulting from the fastest ion of the ejected ion packet.^19^

Hence, all the dissipation errors— not merely those from the waveform defects–should be compensated to retain the ideal motion of each in-trap ion. CY Cheng et al. proposed to preserve the Paul trap scheme governed by a simple Mathieu’s equation for the entire MS scan (Supporting Information Section 1).^17,20,21^ Its dynamical implications require the frequency and amplitude of the sinuous waveform to be constant. Thus, the mass spectrum is derived from a sequence of constant-frequency steps bridged by constant-phase conjunctions (CPCs) (Supporting Information Section 2). The waveform time-out duration with such CPC is designed to be short compared to the period of one sinuous cycle (Figure 1E).

Following the infinitesimal analysis of in-trap motion, the ion’s velocity disturbance introduced by one CPC is linearly proportional to the ion’s instant displacement. Moreover, the ion’s displacement disturbance introduced by one such CPC is smaller in one additional order of magnitude and can be neglected while using CPCs to bridge spectrometry steps (Supporting Information Section 2). Furthermore, along with each constant-frequency step, additional CPCs can be arranged onto the main waveform for phase modulation. Hence, the dissipation error of each ion’s motion can be minimized in the long run (Supporting Information Section 3). This research aims at phase-modulated, stepwise frequency scan QIT spectrometry for high-resolution intact-protein analysis. In this case, the secular oscillation of in-trap motion provides a phase-sensitive routine for high-resolution resonance detection (Supporting Information Section 3).^8^ After the modulation, the in-trap ions of the same *m*/*z* and secular degeneracy are populated in sync. Furthermore, by applying two more auxiliary pulses during each constant-frequency step of the mass scan, their phase, polarity, and in-between period enable the selection of specific ions to be resonantly ejected and detected. In addition, upon resonance, the nonselected ions are kept inside the ion trap and contribute nothing to the spectral line. Thus, high-resolution and high-sensitivity MS detection is attained.

## Experimental Section

### Sample Preparation

Samples were prepared for matrix-assisted laser desorption and ionization (MALDI), a soft ionization technique. The target proteins were cytochrome C (CytoC, MW = 12,384 Da), insulin (MW = 5734 Da), myoglobin (MW ∼ 17 kDa), bovine serum albumin (BSA, MW ∼ 66 kDa), and β-galactosidase (MW ∼ 116 kDa). All protein standards were diluted to produce 100 μL of a 50 μM solution [solvent: H_2_O/ACN/trifluoroacetic acid (TFA), 50:50:0.1] in advance. All chemical reagents and compounds, including acetone, acetonitrile (ACN), sinapic acid (SA), and TFA, were purchased from Sigma-Aldrich (St. Louis, USA). Pure deionized water (18.2 Ω) was sterilized and passed through a 0.22 μm filter to reduce biological contamination.

**Figure 2:**
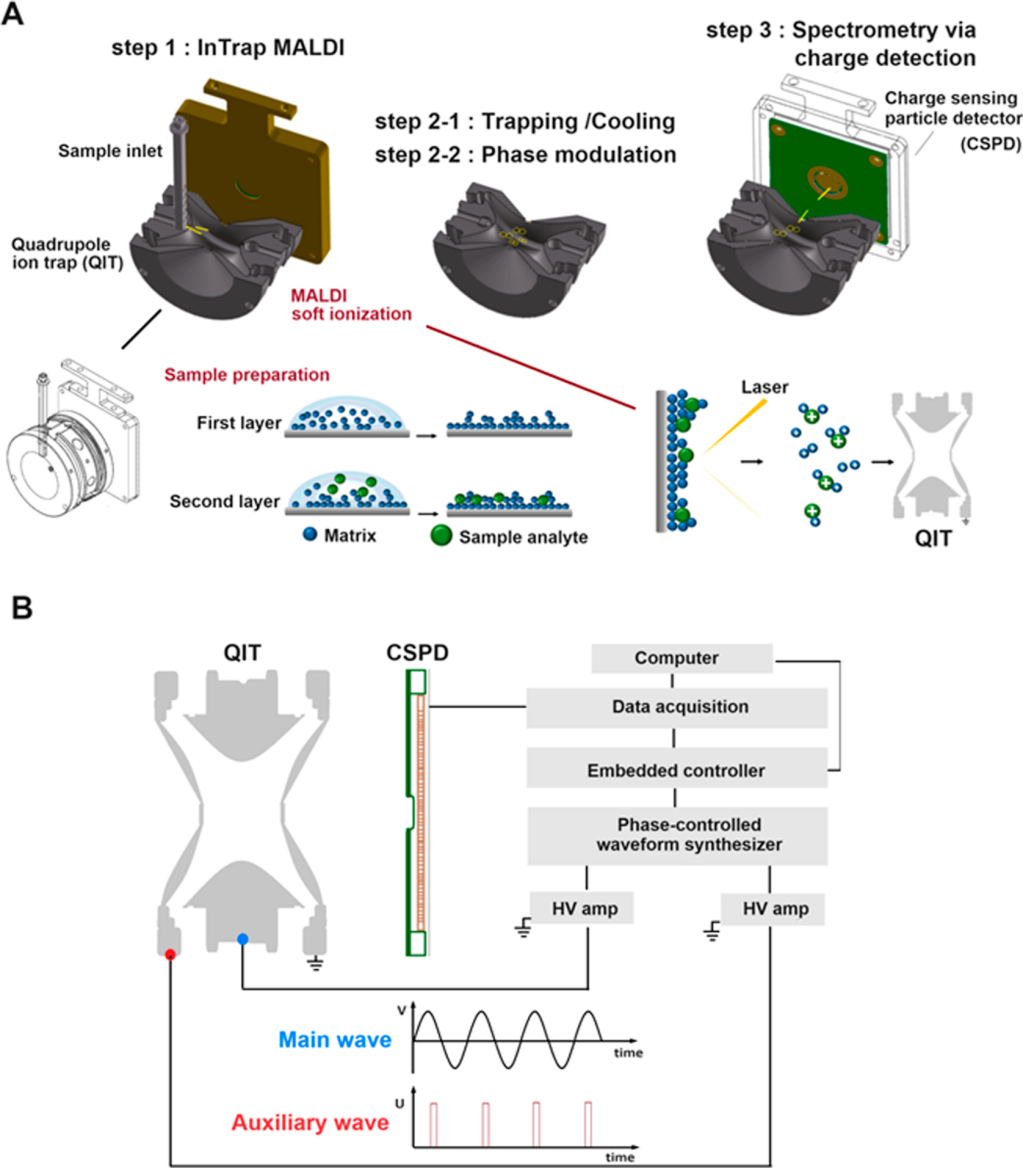
Schematic of the inTrap MALDI mass spectrometer.^17^ (A) inTrap MALDI: assembly, process, and sample preparation. (B) System configuration.

SA, as the matrix for MALDI, was prepared into an oversaturated solution with acetone as the solvent. MALDI samples were prepared via a two-layer method on the stainless-steel surface of the inlet probe. The bottom matrix layer, obtained from a drop of matrix-rich solution (0.6 μL), was quickly dried. Next, the sample layer was obtained from a drop of protein-rich solution (0.6 μL) (Figure 2A), which was also quickly dried to evenly deposit sample molecules over the top surface of the matrix layer (Figure S1).

### Waveform-Programmable MS Platform

MS measurements were conducted on an AMS-200 inTrap MALDI mass spectrometer (Acromass Tech. Inc., Taiwan). Figure 2B depicts the whole assembly, including the embedded platform for data acquisition and waveform drive. The MALDI inlet is embedded within the ring electrode of the Paul trap, leaving one tiny through-hole for incoming laser pulses and outgoing ion packets.

With a charge detector closely attached right outside the end-cap electrode, the Paul trap assembly is designed to operate in a compact vacuum chamber, wherein a steady flow of buffer air is maintained to keep an in-trap pressure of 10 mTorr. The main RF waveform and auxiliary pulses are phase-synthesized by an FPGA-based embedded controller, HV-amplified, and sent to the ring and end-cap of the ion trap (Figure 2B).

### DMZ Stepwise Frequency Scan Mode

**Figure 3:**
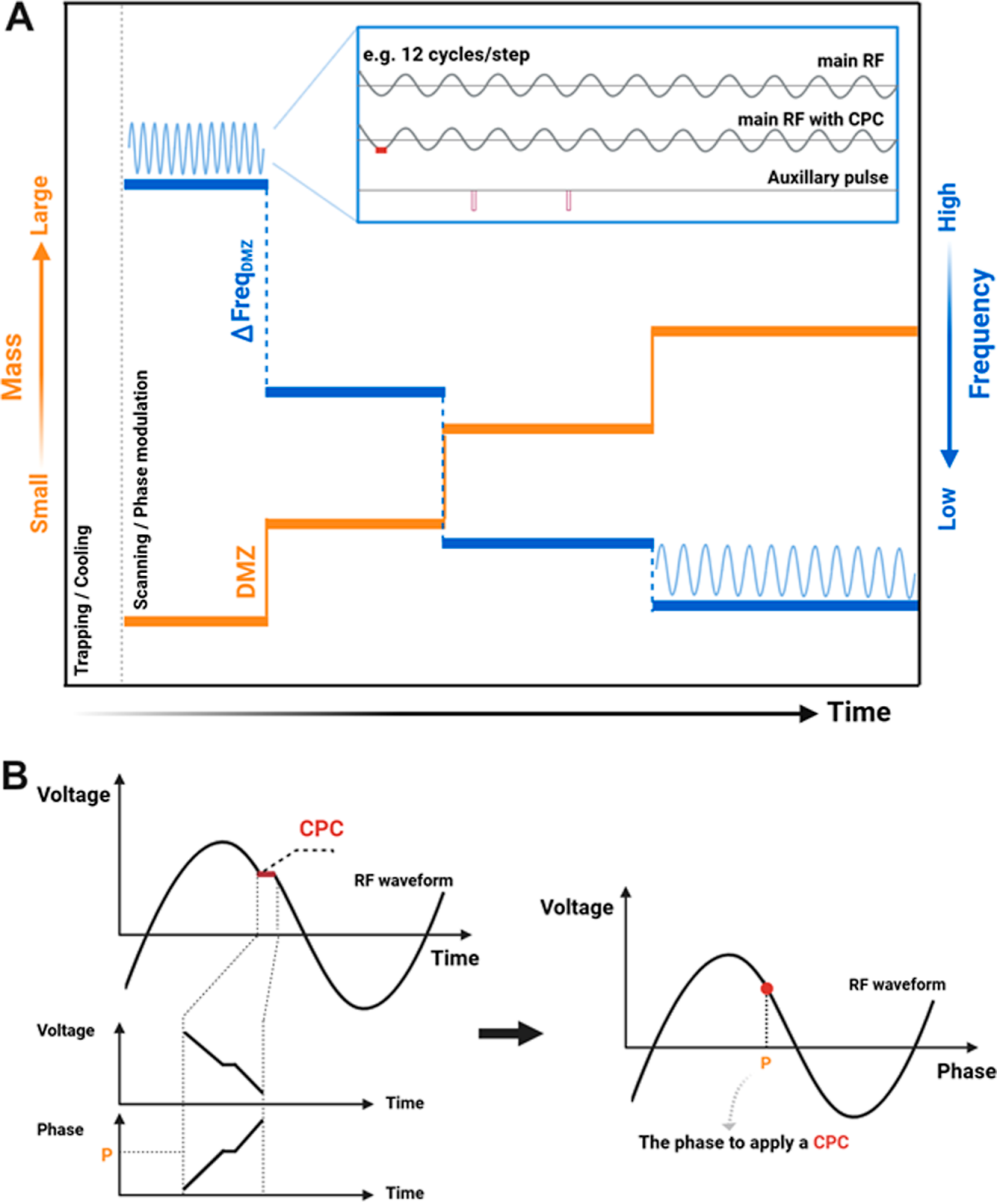
Stepwise frequency-changing MS scan. (A) “Mass vs step” or “frequency vs step” MS scan depictions are presented. In the sequence of constant-frequency steps resulting in an MS scan, each step corresponds to one specific mass (or *m*/*z*). Thus, the MS scan is a sequence of frequency jumps. All jumps correspond to the same difference of *m*/*z* (DMZ) (i.e., the mass spectrum is nominally resolved by the DMZ). (B) The main RF waveform uses one constant-phase conjunction (CPC) to connect the two neighboring steps. The conjunction is devised to be a short time-out in the waveform so that the waveform’s phase and voltage are briefly kept constant. For a waveform in the phase domain, the location for the CPC application is a singular point, introducing almost no dispersive deviation to the in-trap motion (Supporting Information Section 2).

The layout of the main RF waveform is arranged into a sequence of constant frequency steps, with CPCs bridging them (Figure 3A). A CPC lasts 5 ns, much less than 1% of every RF cycle (Figure 3B). For such an MS scan, the waveform in the phase domain is sinuous almost everywhere (Figure 3B). Thus, the CPC introduces almost no dispersive deviation into the in-trap motion (Supporting Information Section 2).

The first step is initiated earlier to capture the MALDI ions with heavier mass than the low mass cutoff, set by the fixed amplitude, the initial frequency, and the instability boundary. Given one fixed voltage amplitude, the conversion of frequency to mass (or *m*/*z*) is based on the location of the stability diagram according to Mathieu’s equation (e.g., Figure S2 and Table S1).

During a mass scan, all frequency jumps correspond to the same difference of *m*/*z* (DMZ), which is the preset or nominal mass resolution. Each step is composed of 12 RF cycles or 2–4 multiples of 12 RF cycles. The duration of 12 (or more) RF cycles is empirically determined to allow, after the frequency jump, those unstable or resonant in-trap ions to be ejected from the ion trap and detected right before the next jump.

### Phase Modulation with Constant-Phase Conjunctions (CPCs)

**Figure 4:**
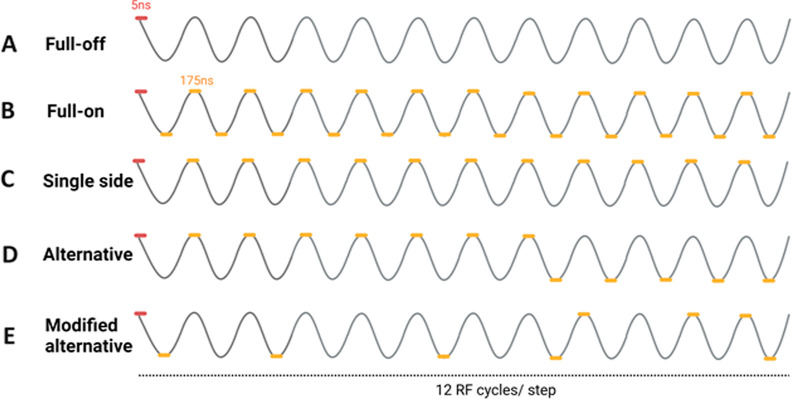
Various arrangements of CPCs in each step were used to test phase modulation. (A) Full-off: no CPCs. (B) Full-on: CPCs at every peak of both polarities. (C) Single-side: CPCs at every peak of one polarity. (D) Alternative. (E) Modified alternative. Each step comprises 12 RF cycles, with 5 ns CPCs for bridging steps. Nevertheless, much longer CPCs (175 ns) were needed for modulation.

Some more regular CPCs are configured into each step of the RF waveform (Figure 4); this is designed to phase-modulate the secular oscillation of in-trap ions, while the secular period is nearly a integer multiple of the RF period. In particular, the secular period is about twice the RF period in the case of the motion of in-trap ions near the boundary of the unstable ejection. Furthermore, for a step with 12 RF cycles, the proper modulation takes effect when the secular resonance degeneracies of in-trap ions are 2, 3, 4, 6, and 12. These degeneracies correspond to the beta values (β) of in-trap motions 1, 2/3, 1/2, 1/3, and 1/6 (Supporting Information Section 2).^11,22^

### Secular Phase-Sensitive Auxiliary Pulses

**Figure 5:**
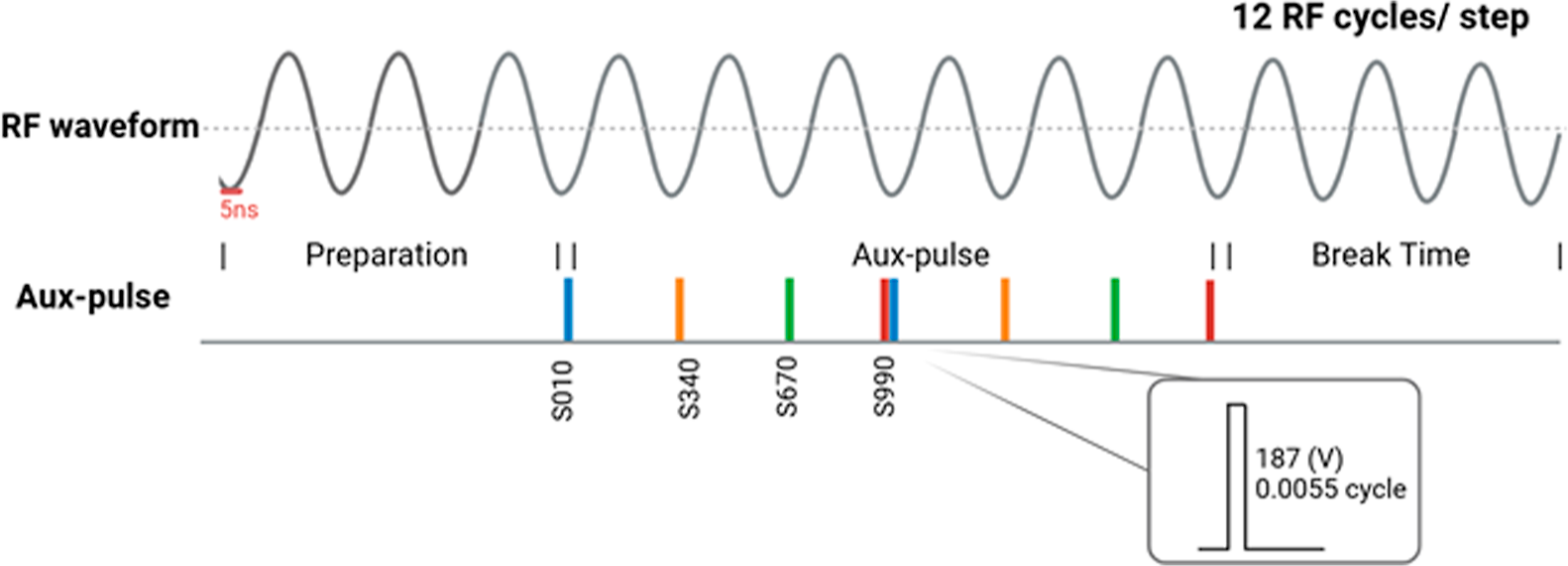
Schematic of the pairs of auxiliary pulses for secular resonance, located at various phases within the steps of the 12 RF cycles. Each pulse is 187 V (height) and 0.0055 RF cycle (width). For example, the notation of pulse S010 is located in the range of 0.0100 to 0.0155 of the RF cycle. Here, the companion pulse of S010 is later separated by three RF cycles corresponding to the secular resonance of β = 2/3. These pulses are located at least three RF cycles behind the bridged CPC to let in-trap ions adapt to a new frequency step. Furthermore, the pulses are located at least three RF cycles before the next bridged CPC to let in-trap ions stay away from the influence of pulses before the frequency jump.

During the mass scan, once the motion of each in-trap ion has been well-modulated, the ions of the same *m*/*z* are populated in a way their secular motion is in sync. Because of the degeneracy by the unstable ejection, additional auxiliary pulses (Aux-pulse) for resonant ejection are recommended.^23^ The electric polarity and the duration between two such pulses select synchronous in-trap ion of specific degeneracy, which are to be resonantly ejected and detected at one particular secular phase (Figure 5). Meanwhile, the remaining in-trap ions of nonresonant degeneracies will stay inside the trap and be detected later via unstable ejection. Various pulse phase locations are set to identify the resonance, considering the degeneracy of secular states and the effectiveness of secular modulation (Figure 5). Here, the β value is 2/3 (i.e., other than 1). A β value of 1 indicates the instability boundary for unstable ejection. On the other hand, a β value of 2/3 corresponds to a secular period of three RF cycles. It is the lowest cycles to exhibit rapid linear resonance, distinct from the high-order harmonic resonances of the in-trap motion.

### Charge-Sensitive Particle Detector (CSPD)

All ejected ions are collected by the charge detector, CSPD, instead of the microchannel plate (MCP). The MCP can be operated only within a costly high vacuum and struggles to collect rare secondary electrons from the dynode, where the heavier and much slower protein ions impinge.^24^ Operating near the ion trap at a pressure of 1 mTorr, the charge detector is designed to be highly sensitive to discrete charge packets yet easily saturated to even a weak continuous current (Figure 2).

The CSPD is a low-noise, low-leakage preamplifier, with a rise time of 0.5 μs and a much longer fall time of 10 ms; the leakage is less than 5 pA and the output noise is 20 mVpp. The CSPD is basically a pole-matched cascade of one transistor (JFET) and one negatively feedback op-amp, in which the feedback is a resistor (10 G Ω) in parallel to a capacitor (1pF), and then in series to a resistor network for current compensation.^17,21,25^ Also, the CSPD can be operated at pressures from 1 atm to 1 × 10^–6^ Torr.

During the MS scan, the ion packets sequentially ejected from the end cap are straightforwardly collected by a metal plate and converted to instant-charging events to modify the voltage difference across the 1 pF capacitor. Subsequently, the voltage profile is acquired and postprocessed into one mass spectrum.

Therefore, the smallest number of RF cycles in each constant-frequency step is set according to the rise time (approximately 0.5 μs) and the flight time of the ion packet (less than 0.05 ms). Here, for instance, the 12 RF cycles per step were suitable for detecting protein ions of 1–100 kDa.

### Signal Postprocessing and Spectrum Structure

The output voltage profile (i.e., the raw data) is transformed back to the input current profile according to CSPD’s voltage-to-current response. In this way, the long-tail feature of the voltage signal can be rectified. Subsequently, discrete charging events are highlighted by the edge correlation of the Lorentzian function, which profiles the ion packet.

Here, one denoise convolution was needed to suppress fast electronic fluctuations. Next, the data string was nonlinearly amplified to the power of 6 or more. This operation for contrast enhancement was akin to the multiple stages of charge amplification in the electron multiplier tube or array. Furthermore, the data string was renormalized with respect to the maximal peak for each mass scan. The final mass spectrum, expressed as “intensity (in arbitrary unit) vs mass (in *m*/*z*),” was composed of at least 50 scans to manifest the system’s overall dissipation. Mass spectra were plotted and labeled using MATLAB and Origin graphic tools.

## Results and Discussion

The inTrap MALDI mass spectrometer has three novel elements that allow obtaining high-resolution intact-protein mass spectra: (1) the MS scan based on a stepwise sequence of CPC-bridged, constant-frequency steps; (2) the phase modulation via additional CPCs per step in longer secular periodicity; (3) the detection to precisely select one secular resonance without degenerate ambiguity.

**Figure 6:**
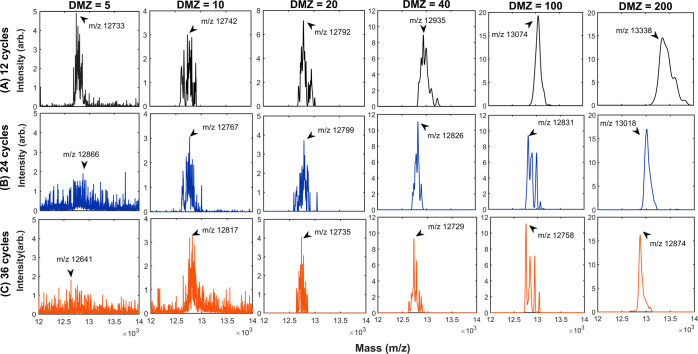
MS measurements of the simple CPC-bridged DMZ stepwise frequency scan were obtained by detecting the unstable ejection. The scan process is a sequence of constant-frequency steps. Each frequency jump is bridged by one CPC, with a frequency change corresponding to the mass difference (DMZ). There are three options for the RF cycles per step: 12, 24, and 36, and six options for the DMZ: 5, 10, 20, 40, 100, and 200 (Da). Each final mass spectrum, expressed as “intensity (in arbitrary unit) vs mass (in *m*/*z*),” is composed of 50 scans to manifest the system’s overall dissipation.

A simple CPC-bridged, stepwise frequency scan was devised to detect the unstable ejection yielding the MS measurement. The intact CytoC protein ions were the target, given the mass range of 12–14 kDa. As depicted in Figure 3A, the size of the mass jump (DMZ) determines the steps and timing of the MS scan. Figure 6 depicts the mass spectra obtained by applying various DMZs and RF cycles per step. A short-duration CPC introduced no unexpected MS effects according to Mathieu’s scheme of ion trap dynamics (Supporting Information Section 2).

Figure 6 shows the location of the spectral peak shifts from 13,338 Da of DMZ = 200 to 12,792 Da of DMZ = 20, and then to 12,742 Da of DMZ = 10. The observed trend agrees with previous reports. Furthermore, given the fixed RF cycles per step, as the DMZ becomes smaller, the spectral line weakens but with a more fine-resolved structure. Thus, the smaller the DMZ, the smaller the number of unstable ions ejected in each frequency jump, and the more likely for ions to be completely detected in the constant-frequency step. Likewise, given the same DMZ, the spectral line shows a better fine-resolved structure for the option with more RF cycles per step.

Along with the measurement statistics of the final mass spectra, dissipation was detected by inspecting the spectral shift and spread. In particular, given a DMZ that yields an adequate number of ejected ions, dissipation is evident whenever the spectral fine structure is not DMZ-by-DMZ-resolved (i.e., not fully fine-resolved). As the DMZ decreases, the spectral line shifts earlier and becomes narrower (Figure 6). Thus, if the number of steps in one mass scan increases, dissipation decreases. However, as the number of RF cycles per step doubles or triples, dissipation increases. This outcome indicates that the accumulation of dissipation, along with the “free-running” (or constant-frequency) RF waveform, could be interrupted by the bridging CPCs (Supporting Information Sections 1 and 2).

**Figure 7:**
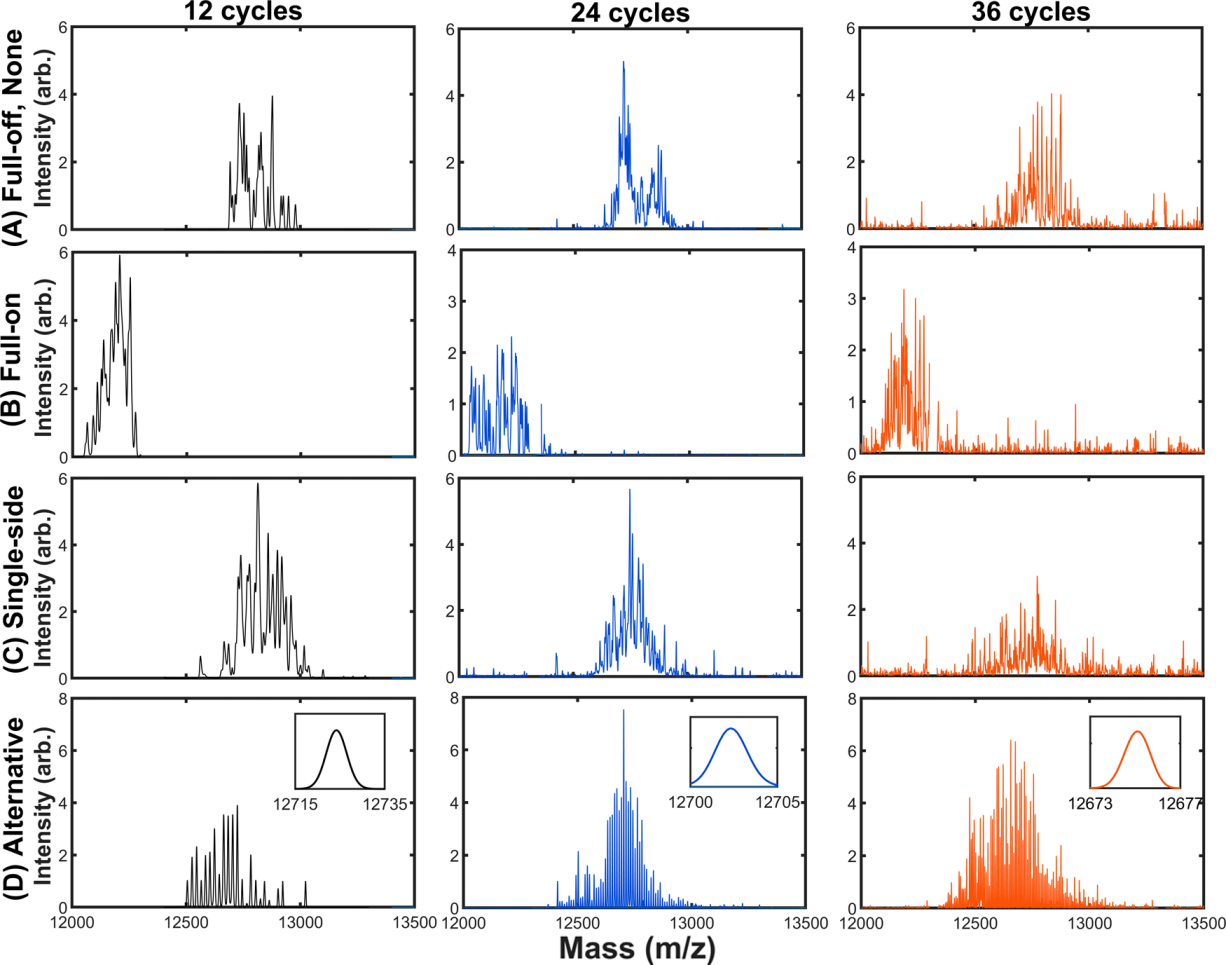
MS measurements of phase-modulated, CPC-bridged stepwise frequency scan by detecting the unstable ejection. In contrast to the unmodulated scan, three CPC patterns are arranged in each step to modulate the secular oscillation of all in-trap ions. Each final mass spectrum is composed of 50 scans to manifest the system’s overall dissipation.

Next, additional CPCs were introduced into each constant frequency step for modulation. Figure 7 depicts the spectral results of applying the first four different arrangements in Figure 4. As a result, a notable spectral line shift was detected between the “full-off” and “full-on” options (Figure 7A,B). Nonetheless, the result was indifferent to the “full-off” and “single-side” options (Figure 7A,C). On the other hand, only the “alternative” option resulted in a fully fine-resolved structure, in which each fine peak had the same nominal resolution of DMZ (Figure 7D). This result suggests that the asymmetry of the alternating CPC pattern is crucial for modulation. That is, modulation can be justified to have a high-resolution spectrum which preserves Mathieu‘s scheme of ion-trap dynamics.

**Figure 8:**
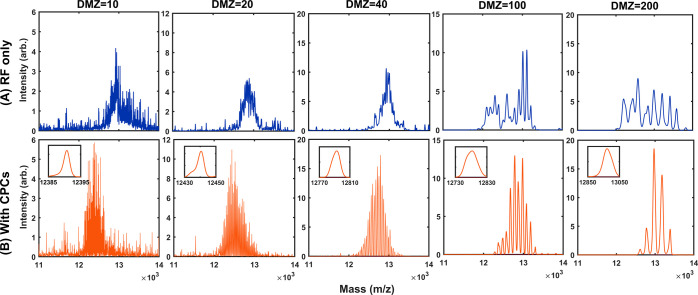
A comparison of simple vs modulated MS measurements, obtained by CPC-bridged stepwise frequency scans that detect the unstable ejection. The modulation pattern was modified so that the micro-oscillation periodicity was not introduced. Each final mass spectrum is composed of 50 scans to manifest the system’s overall dissipation.

For justification, the “modified alternative” option was devised for a modulating periodicity of 12 RF cycles. The location of additional CPCs among these 12 RF cycles was arranged so they were alternating without the periodicity of the micro-oscillation (Figure 4E). Thus, the spectral line was modulated to be with a fully fine-resolved structure, along with a mild line shift (Figures 7D and 8B). By contrast, the remarkable line shift observed in the “full-on” option was attributed to a coincident periodicity of the modulation and the micro-oscillation, altering the primitive dynamics of in-trap ions (Figures 4B and 7B). In addition, the “single-side” option barely modulated the result because it was made of only nonalternating CPCs for bridging (Figures 4C and 7C).

Thus, a stepwise frequency scan (Figure 4E) with the “modified alternation” option reveals a spectral contrast as a result of the proper modulation introduced by additional CPCs (Figure 8A vs B). The resolution is enhanced when the DMZ is smaller. However, the spectral line is DMZ-by-DMZ-resolved only after the modulation. That is, for each mass scan, the dissipation exerted upon the in-trap ions is modulated to a minimum. Thus, all unstable in-trap ions are DMZ-by-DMZ-resolved, ejected in sync, and detected. Here, the spectral line of CytoC is centered at 12,440 Da (Figure 8B, DMZ = 20), near the conventional 12,384 Da.

The results depicted in Figure 8B demonstrate a substantial improvement over previous ion trap MS approaches requiring prefragmentation for masses exceeding 12 kDa. The peak range and ionization are also similar to published results using MALDI-TOF.^26−29^ The spectral line characteristics resemble those of electrospray ionization, but most of the charged ions are [M + H]^+^ rather than multiple peaks with different charges coexisting in the spectrum.^30,31^

Nevertheless, the DMZ-resolved structure was nearly 1 kDa full width at half-maximum (fwhm), revealing inhomogeneity derived from the real ion trap. The motion of in-trap ions exhibits a radially off-center deviation and is split over a nonlinear field, suggesting a degeneracy splitting in the secular degree of freedom. Hence, the detection via unstable ejection allowed all instabilities resulted in the MS errors as shown in Figure 8B.

Therefore, to obtain a “clear-cut” and narrower MS spectrum, auxiliary pulses are introduced for the detection via resonance of in-trap ions at a specific secular state. That is, along with a valid modulation, in-trap ions of the same *m*/*z* and the same secular phase are expected to be ejected by applied pulses at an earlier and specific constant-frequency step, rather than finally leaving the trap via unstable ejection.

**Figure 9:**
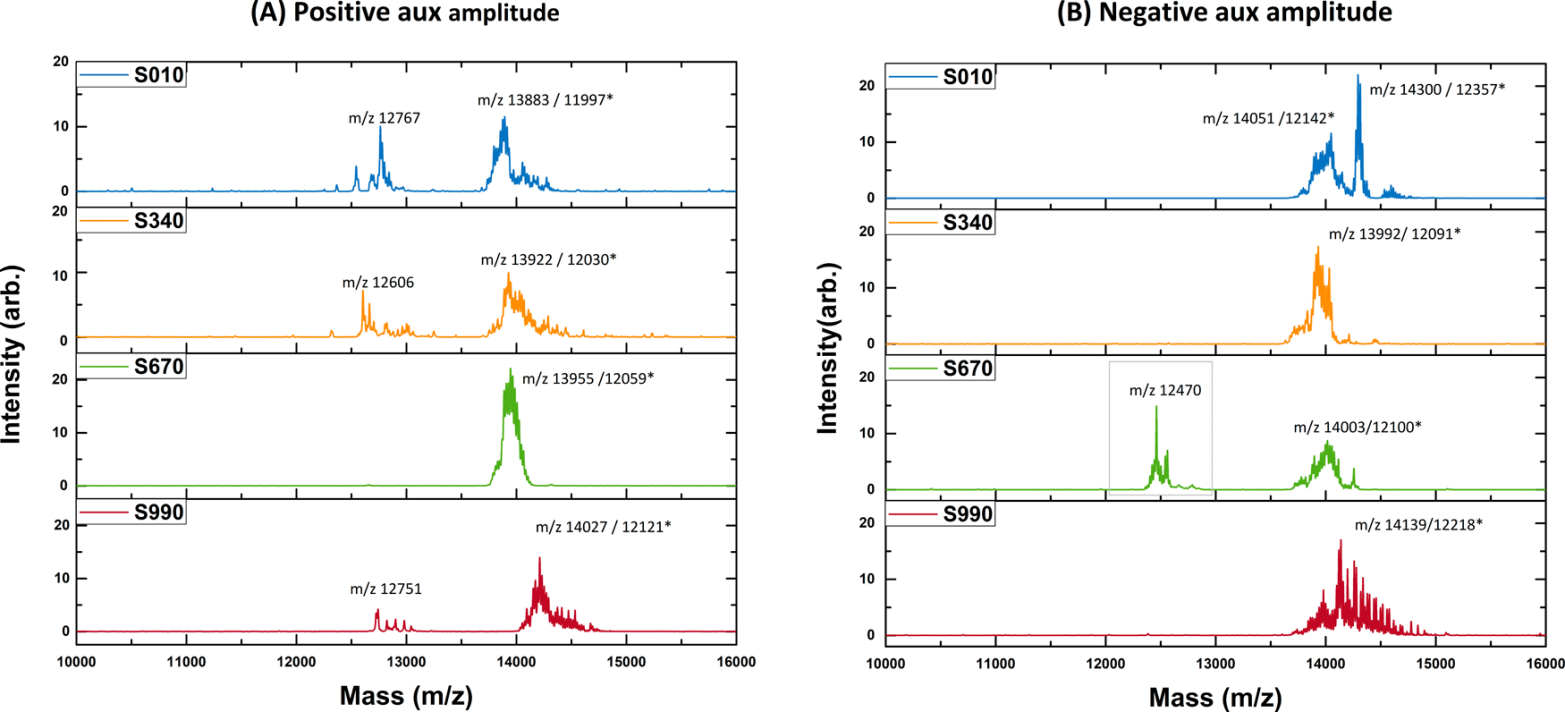
MS measurements of the phase-modulated, CPC-bridged stepwise frequency scan were obtained by detecting the resonant ejection for β = 2/3. The resonant line is located well before the spectral line because of the unstable ejection. The modulation pattern was modified to avoid introducing the micro-oscillation periodicity. Two auxiliary pulses (pulse height = 187 V, pulse width = 0.0055 RF cycle, separated by three RF cycles) were applied to scan specific phases to detect the secular resonance (Supporting Information Section 3). Three resonant phases were identified accordingly. Upon identifying these resonant phases, a singlet and a doublet were confirmed via pulse polarity (Figure S4).

Figure 9 depicts the spectra modulated via the β = 2/3 resonance. Two auxiliary pulses were applied at specific secular phases to trigger resonant ejection (Figure 5). Once one of the resonant phases was found, using the correct polarity, the other two could be accurately identified. For the MS measurement, the β = 2/3 resonant lines were identified as a singlet and a doublet. The main spectral line of the singlet was located at 12,470 Da (DMZ = 20 Da). This outcome demonstrates the molecular signature of CytoC (Figure 9B), with system degeneracies being phase-selectively suppressed.

**Figure 10:**
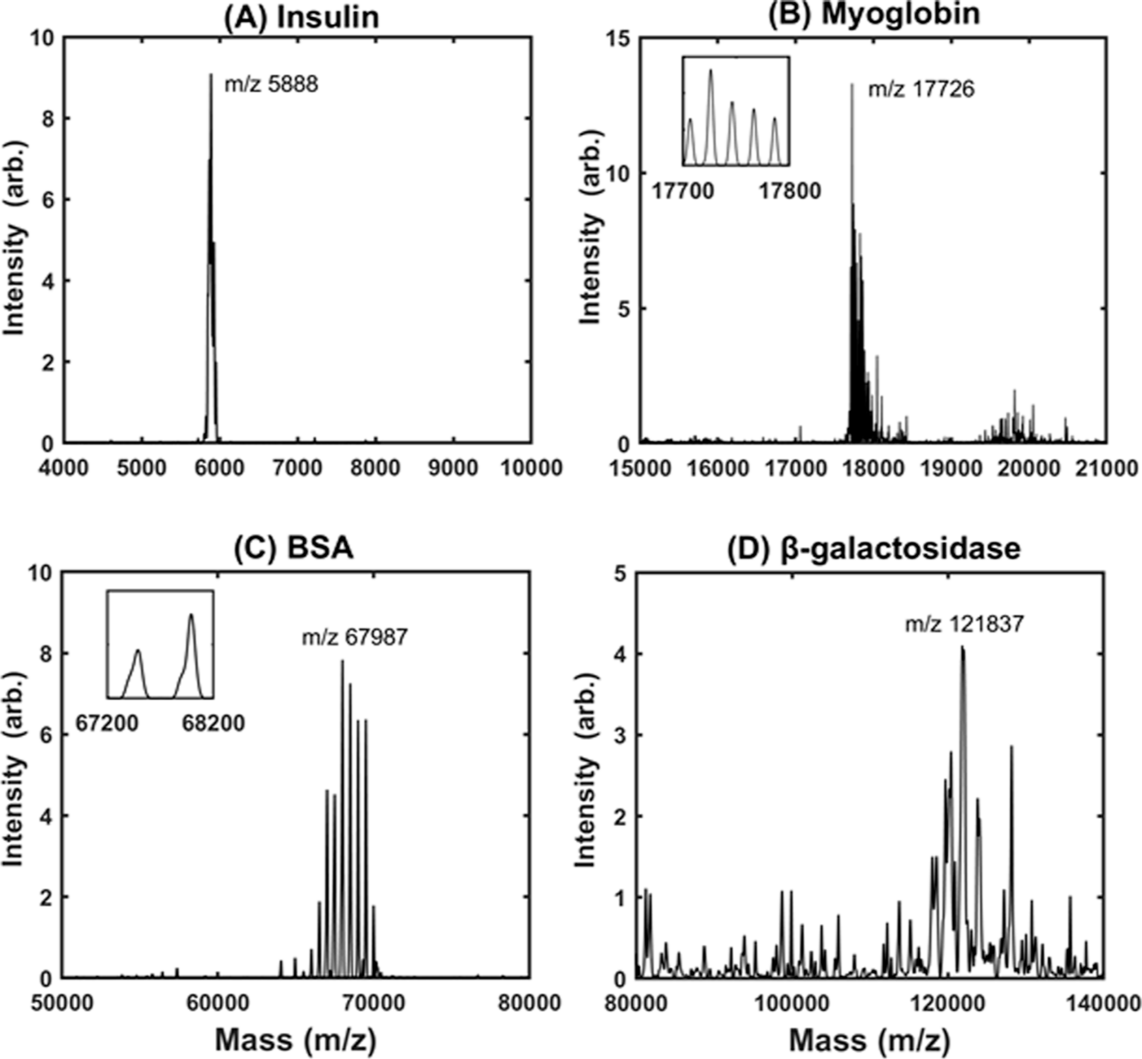
Quick MS results obtained for insulin, myoglobin, BSA, and β-galactosidase. Standard proteins were detected using the inTrap MALDI MS. The maximum peaks were as follows: (A) insulin, 5888 Da; (B) myoglobin, 17.7 kDa; (C) BSA, 67.9 kDa; (D) β-galactosidase, 121.8 kDa. The stepwise MS via unstable ejection and secular modulation reduced dissipation. Despite residual fluctuations from the splitting of secular degeneracy and system defects, the quick MS scans of these proteins resulted in fine-resolved spectra and a good signal-to-noise ratio, which helped attain the correct secular phase to obtain the molecular signature.

Altogether the three MS implements: constant-frequency steps, main modulation, and auxiliary pulses, the technique based on CPCs could resolve the spectral line without ambiguity. Even without auxiliary pulses, the first two implements may be used for a quick high-resolution MS scan of the protein ions in the inTrap MALDI instrument, following the top-down approach for intact protein analysis. Figure 10 depicts the quick spectral results obtained for insulin, myoglobin, BSA, and β-galactosidase. These proteins are all ionized intact and trapped inside this compact MS assembly. Then, they are secularly modulated, scanned in a stepwise manner, and detected via unstable ejection.

According to the MS setting for CytoC, DMZ = 20 is optimal to achieve an adequate signal-to-noise ratio and high resolution for a stepwise scan of masses ranging from 2 to 20 kDa. The outcome for insulin and myoglobin is depicted in Figure 10A,B. Moreover, DMZ = 500 is adequate for BSA, with a mass of around 60 kDa (Figure 10C). In addition, DMZ = 1000 is adequate for β-galactosidase, which is composed of units with masses over 100 kDa (Figure 10D). Ion packets can be detected via unstable ejection because the ionization efficiency of the target proteins keeps the DMZ small. As long as the number of target ions is sufficient, the DMZ can be reduced and the resolution can be enhanced.

## Conclusions

A top-down MS approach was used for intact protein analysis. The high-resolution signature of cytochrome C (CytoC) molecules was obtained via a QIT system. Most target molecules can be ionized and caught undamaged inside the ion trap of this compact MS assembly. The electron multiplier is not used to gather secondary electrons from ejected ions that impinge upon the dynode. Instead, the charge detector is used to register every ion packet ejected during the MS measurement.

In summary, three innovations have been presented:

(1) for a typical Paul trap, the MS process is devised to preserve the stability diagram governed by Mathieu’s equations. Thus, the RF waveform used to manipulate in-trap ions is divided into a sequence of constant-frequency steps, interconnected by a short time-out. Such a constant-phase conjunction for bridging two steps introduces the least dispersion to the mass conversion scale.
(2) The conjunction results in a displacement variation, linear to the velocity of the in-trap ions. Thus, some conjunctions can be arranged into each step to modulate the secular motion of in-trap ions and compensate for the disturbances from the ion trap. Along with the modulation, the fine-resolved spectral line has shown the attained resolution set by the MS measurement.
(3) Two auxiliary pulses were introduced in each step of the MS measurement to get rid of split degeneracy from the unstable ejection. In this way, phase-modulated ions of a specific secular-state can be selected, allowing them to be resonantly ejected and synchronously detected as one spectral line. By contrast, other ions not correlated in the right phase remain in the trap until they reach the instability boundary (i.e., unstable ejection).

Therefore, high-resolution intact protein analysis is possible using the MS assembly comprising MALDI, a QIT, and a charge detector. Thus, the biomolecular signature (the chemical structure in its natural state) can be obtained with the smallest dispersive error and without extra calibration. The same considerations apply to the Q-ToF system with soft ionization.

## Supporting Information Available

Additional experimental information includes an introduction and description of the dynamics of the in-trap ion motion, additional results such as two-layer sample preparation and MS analysis with resonance ejection, and experimental details. All Supporting Information was merged into the same document (PDF)
