# Supplementary material for: High-Resolution Intact Protein Analysis via Phase-Modulated, Stepwise Frequency Scan Ion Trap Mass Spectrometry
Source: Anal Chem. 2024 Sep 6;96(37):14867–76. doi: 10.1021/acs.analchem.4c02775 (PMC11411492; doi:10.1021/acs.analchem.4c02775)
Supplement: Supplementary file 1 — ac4c02775_si_001.pdf [file ac4c02775_si_001.pdf]

Supplementary Information

High-Resolution Intact Protein analysis via Phase-Modulated, Stepwise Frequency-Scan Ion-Trap Mass Spectrometry

Fang-Hsu Chen[1], Chun-Yen Cheng[1&2], Szu-Wei Chou[2], Cheng-Han Yang[3], I-Chung Lu[3], Ming-Long Yeh*[1&4]

[1]Department of Biomedical Engineering, National Cheng Kung University, Tainan City 701, Taiwan

[2]AcroMass Technologies Inc., Hukou, Hsinchu, 30352, Taiwan

[3]Department of Chemistry, National Chung Hsing University, Taichung City 40227, Taiwan

[4]Medical Device Innovation Center, National Cheng Kung University, Tainan City 701, Taiwan

Table of contents

Supplementary Sections **3**

1. Dynamics of In-Trap Motion: Mathieu's Scheme in the Phase Domain. 3
2. Dynamics of In-Trap Motion: Constant-Phase Conjunction to Bridge Constant-Frequency Steps. 4
3. Dynamics of In-Trap Motion: Using CPCs to Achieve Secular Modulation. 6

Supplementary Figures **8**

Figure S1. Distribution of analytes: single-layer method versus two-layer method......... 8

Figure S2. Stepwise frequency changes for two mass regions. 9

Figure S3. Schematic of the motion of an in-trap ion and three possible phase alignments for the effectively modulated motion of in-trap ions. 10

Figure S4. MS measurement used to probe resonance ejection. 11

Supplementary Tables **12**

Table S1. Conversion table of ion mass and waveform frequency, given a DMZ, for β=1 the state to initiate unstable ejection. 12

Table S2. Number of steps near an integer degeneracy under different DMZs. 13

Supplementary Sections

1. Dynamics of In-Trap Motion: Mathieu's Scheme in the Phase Domain.

The dynamic equations governing both the in-trap axial and radial motions, driven by a simple sinusoidal wave, are described by Mathieu's differential equation in the time domain. This in-trap motion maintains a constant angular momentum around the trap axis of symmetry. In the radial equation, a "centrifugal force" term is neglected for ions with zero initial azimuthal speed. In reality, the ion trajectory circles around an off-radial-center axis and never passes through the trap axis of symmetry (**Figure S3A**). For the equations, see (2.60) and (2.61) of "Quadrupole Mass Spectrometry and Its Applications," P.H. Dawson (ed.), Elsevier 1976.

The stability diagram derived from two Mathieu's equations requires the driving RF waveform to have a fixed amplitude, frequency, and DC offset. The diagram location determines the orbit instability for in-trap ions of specific *m*/*z*. With the assumption of a closed orbit for the in-trap motion, the stability diagram includes two beta coordinates (i.e., β_z, β_r) to characterize the state of secular oscillations, both axially and radially.

For cases in which the mass spectrometry (MS) process involves varying the frequency of the driving RF waveform, similar to a "frequency scan," the stability diagram and its secular coordinates are expected to deform, as the dynamics in the time domain no longer fit Mathieu's equation. Consequently, stable MS measurements must be externally calibrated using reference molecules, with the assumption that the dynamics are adiabatically disturbed and do not considerably deviate from the ideal described by Mathieu's equation as below (Eq. S1).

Ideal Mathieu's equation in perfect electric field:

$$\frac{d^2u}{d\xi^2} + [a_u + 2q_u \cos(2\xi)]u = 0 \qquad \text{(Eq.S1)}$$

where u represents the displacement of in-trap motion, 2ξ is the phase angle of RF-waveform, and a, q are respectively the DC and AC parameters.

The dynamics of in-trap ions explicitly depends on the phase of the driving RF waveform. Given a fixed AC amplitude and DC offset, with the phase as the independent variable, both the axial and radial equations of motion remain similar to Mathieu's equation in the phase domain. However, both equations include an additional "driving" term owing to a dispersive coupling between the "velocity" of the in-trap ion and the "frequency variation" of the RF waveform. Accordingly, the differential equation of in-trap motion is as below:

Non-ideal Mathieu's equation revised with real perturbations:

$$\frac{d^2u}{d\xi^2} + [a_u + 2q_u \cos{(2\xi)}]u = \bar{b} \qquad \text{(Eq.S2)}$$

wherein the homogeneous part is sinuously driven in the phase domain, similarly formulated to Mathieu's equation in the time domain. The inhomogeneous part ($\bar{b}$) contains all the above-mentioned non-ideal terms, owing to field defects, buffer gas collisions, ion-ion interactions, and dispersive coupling.

In the phase domain, the "frequency variation" of the RF waveform can be understood and investigated as a definite source of deviations from the stability diagram derived by Mathieu's equation. Other non-ideal but real perturbations caused by high-order electric fields, buffer gas collisions, and ion–ion interactions contribute significant errors and uncertainty to the MS measurement of in-trap ions.

2. Dynamics of In-Trap Motion: Constant-Phase Conjunction to Bridge Constant-Frequency Steps.

As analyzed above, the "frequency scan" in the phase domain introduces dispersive coupling, which tends to distinctly distort the stability diagram. For small molecules, the same MS approach of a slow, continuous frequency scan can be used with the aid of external calibration. However, for much heavier and more complex molecules, a stepwise frequency-

scan approach is devised. In this MS sequence, the waveform frequency is set to a constant value corresponding to one specific *m*/*z* for each step. During such a "constant-frequency" step, the in-trap motion aligns with the stability diagram derived from Mathieu's equations. Consequently, all in-trap ions can be accurately assessed for instability after a reasonable frequency jump to the next step.

A crucial design element for a reasonable frequency jump is using the constant-phase conjunction (CPC) to smoothly bridge the RF waveform between two steps of different frequencies. The CPC is introduced into the waveform for a precise frequency jump, preventing abrupt distortion of the stability diagram. A CPC involves a very short "time-out" of the RF waveform (**Figure 3**), which, in the phase domain, consists of two nearly opposite instantaneous frequency transitions occurring just before and after a specific phase. These two dispersive perturbations scarcely distort the stability diagram because they are designed to nearly cancel each other out to the lowest order in the phase domain. Therefore, with CPCs properly implemented to bridge all constant-frequency steps, the MS path in the stability diagram proceeds almost without dispersive deviation.

Below is to evaluate the influence on the in-trap oscillations, whenever such a CPC is applied. Following the integration of the differential equation, Eq. S2, infinitesimally near the phase to apply CPC, we have two implications: (A) To the first order, the ion's velocity disturbance introduced by one CPC is linearly proportional to the ion's instantaneous displacement. The dispersive contribution of such a CPC of the lowest order, is to the second order (Eq. S3, as below). (B) To the second order, the ion's displacement disturbance introduced by one CPC is much smaller than the displacement itself (Eq. S4, as below).

Below is the influence on the in-trap oscillations whenever such a CPC is applied.

$$\left.\frac{\delta u}{\delta\xi(t)}\right|_{\xi_j^-}^{\xi_j^+} = -\left({\xi_j}^+ - {\xi_j}^-\right)\cdot\left[a - 2q\cdot\cos\left(2\xi_j\right)\right]\cdot u + O\left(\left({\xi_j}^+ - {\xi_j}^-\right)^2\right)$$

(Eq. S3)

$$u\big|_{\xi_j^-}^{\xi_j^+} = O\left(\left({\xi_j}^{+} - {\xi_j}^{-}\right)^2\right)$$

(Eq. S4)

where u represents the displacement (either axial or radial); $2\xi_j$ is the phase at which one CPC is applied; and a and q are respectively the DC and AC parameters in Mathieu's equation, which determine the coordinates of the stability diagram. In particular, $2\xi(t)$ denotes the phase of the simple sinusoidal wave, synthesized as a function of time by the embedded system of the in-trap MALDI mass spectrometer.

Thus, when one CPC is used to bridge the waveform between MS steps, the in-trap motion is perturbed to exhibit a slight velocity variation that is proportional the instantaneous displacement, while the displacement variation remains almost zero. That is, CPCs perturb in-trap ions, and preserve them inside the trap.

3. Dynamics of In-Trap Motion: Using CPCs to Achieve Secular Modulation.

In a prolonged MS scan, dissipation and fluctuations hinder the preparation of a ”state-specific“ packet of ions from the in-trap ion cloud. Nonetheless, the proportional linearity in property (A) allows for the realization of secular-phase modulation through additional CPCs, provided that the modulation periodicity is longer, far different from the periodicity of micro-oscillation.

According to the formula Eq. S3, the velocity variation of in-trap ions is trivial, whenever their displacement is right at zero as the CPC is applied. Otherwise, the velocity of in-trap ions will change a bit, as the CPC is applied. Therefore, when the secular motion of in-trap ions is nearly coincident to the alternating periodicity of applying CPCs, their motion will be “modulated”. And, in the long run, their secular displacement “zeros“ can be gradually synchronous to each other as if those CPCs are trivially applied. As for those in-trap ions with secular motion far different from the alternating periodicity, no modulation will happen since CPCs are effectively applied at random.

Specifically, for the detection of ejected ions from the end-cap electrodes, the instantaneous axial displacement of in-trap ions would be gradually reduced to near-zero, periodically aligning with certain effective phases whenever alternating CPCs are applied secularly. Consequently, in-trap ions of the same *m*/*z* will eventually have their motion modulated to the same secular phase, allowing them to be synchronously ejected in the axial direction.

Given that additional CPCs are arranged in one step of 12 RF cycles (**Figure 4D** or **4E**), in-trap ions of the same *m*/*z* will have their motion effectively modulated while scanning around the constant-frequency steps corresponding to β = 1/6, 1/3, 1/2, 2/3, and 1. Near these steps, the period of the driving RF waveform is nearly matched in integer multiples to the secular periodicity of in-trap motion. In contrast, for steps that do not coincide with these "integer" values, their modulation becomes almost random and thus trivial.

Furthermore, whenever the in-trap motion is effectively modulated, its state transitions into one of the secular degeneracies over the long term. The integer periodicity ratio of secular oscillation to micro-oscillation defines secular degeneracy, which is $2/\beta$ (**Table S2**). For example, with modulation at β = 2/3 (**Figure S3**), there are three possibilities for aligning the primary zeros of in-trap motion concerning the phase of applying an effective "CPC." Notably, two of the three primary zeros always exhibit opposite polarity trends compared with the third zero.

In the MS scan, once the motion of all in-trap ions has been effectively modulated, the secular-phase synchronization of ion-trap motion provides a phase-sensitive routine for high-resolution resonance detection. Additionally, as illustrated in **Figure S3**, two auxiliary pulses at specific phases are employed to quickly trigger resonance ejection. The correct phase for resonance is closely related to the "effectiveness" of secular modulation. **Figure S4** illustrates the results of phase probing. In this case, a sinusoidal wave is not used for the auxiliary resonance, as it is too slow and thus disrupts the secular modulation.

Supplementary Figures

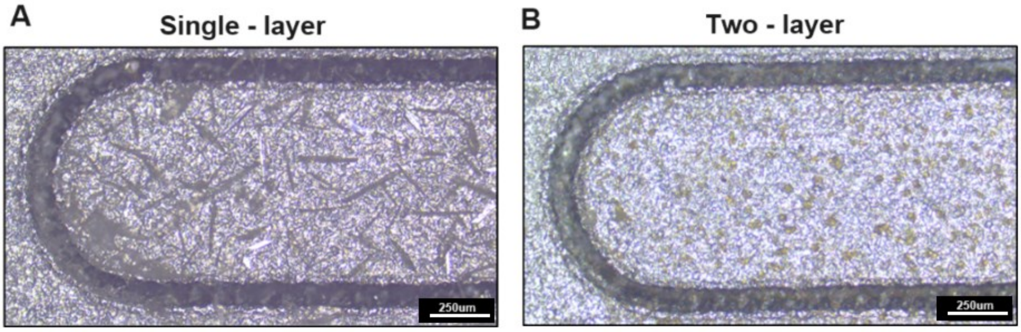

Figure S1. Distribution of analytes: single-layer method versus two-layer method.

(A) Single-layer method: The solution of SA (the matrix) and CytoC (the analyte) is directly applied and dried on the sample probe. This method shows a poor distribution of analytes among the aggregated matrix grains on the plate. The phase rule for two or more components does not guarantee a single-phase solidification. (B) Two-layer method: The first layer is dried from several drops of a matrix-saturated solution. For the second layer, another drop of the analyte solution is quickly dried on top. This method results in a uniform deposition of analytes over the matrix base. The length of the scale bar is 250 μm.

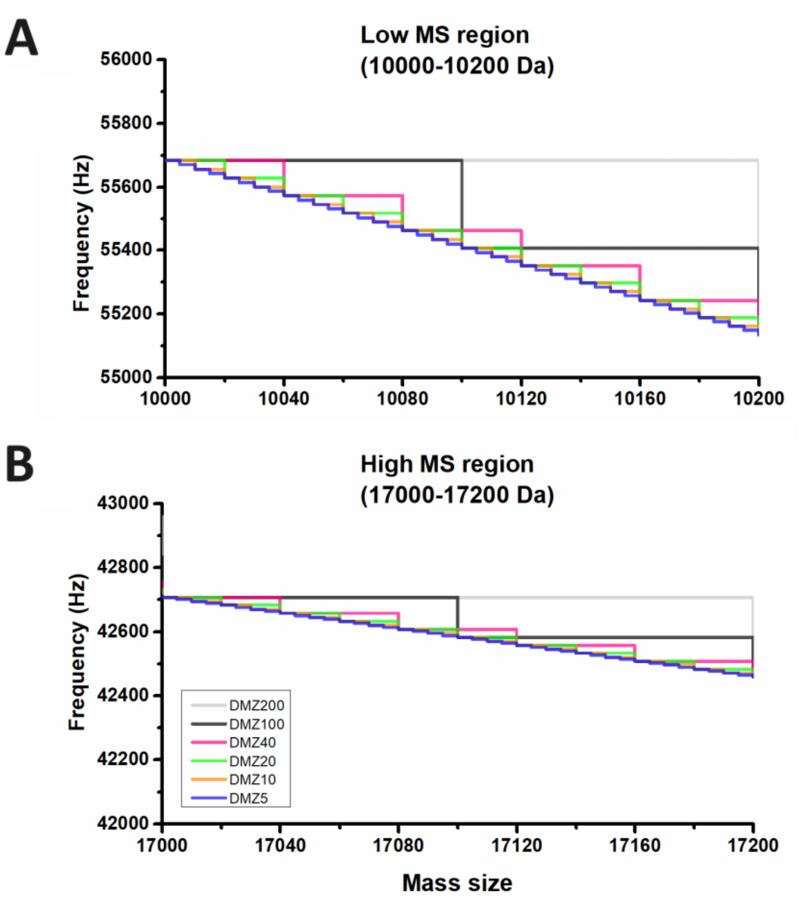

Figure S2. Stepwise frequency changes for two mass regions.

Given the same DMZ, the frequency jump is significantly larger for the small mass range. The frequency conversion is detailed in **Table S1**.

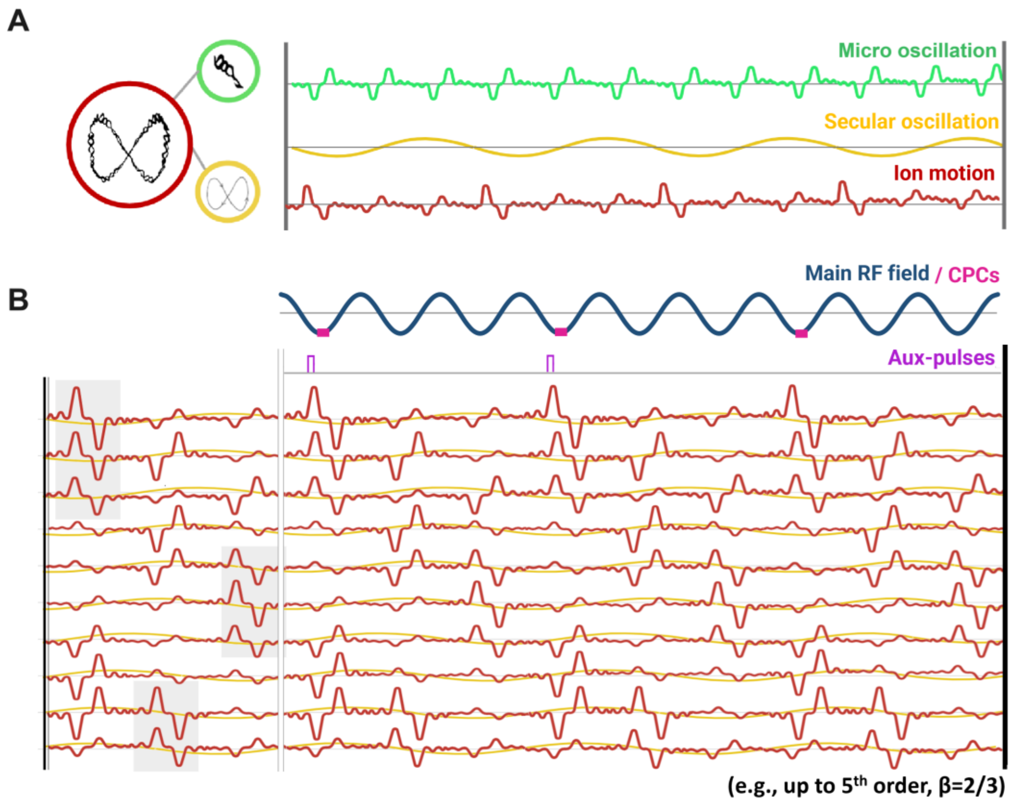

Figure S3. Schematic of the motion of an in-trap ion and three possible phase alignments for the effectively modulated motion of in-trap ions.

The trajectory of an in-trap ion, illustrated in the axial degree of freedom. The ion motion results from the combination of micro-oscillation and secular oscillation. In the case of $\beta = 2/3$, the in-trap motion is characterized by three primary zeros, with significant zero-crossings. (B) The diagram illustrates the phase alignment for the axial motion of multiple in-trap ions after the ions of the same m/z have been modulated into the secular state of $\beta = 2/3$. The ions are aligned with respect to the effective CPCs, as indicated on the RF waveform. This alignment presents three possibilities for the primary zeros of axial oscillation. Two auxiliary pulses, with the appropriate magnitude, are positioned at the correct phase to excite the secular singlet, rather than at the other two phases for the secular doublet.

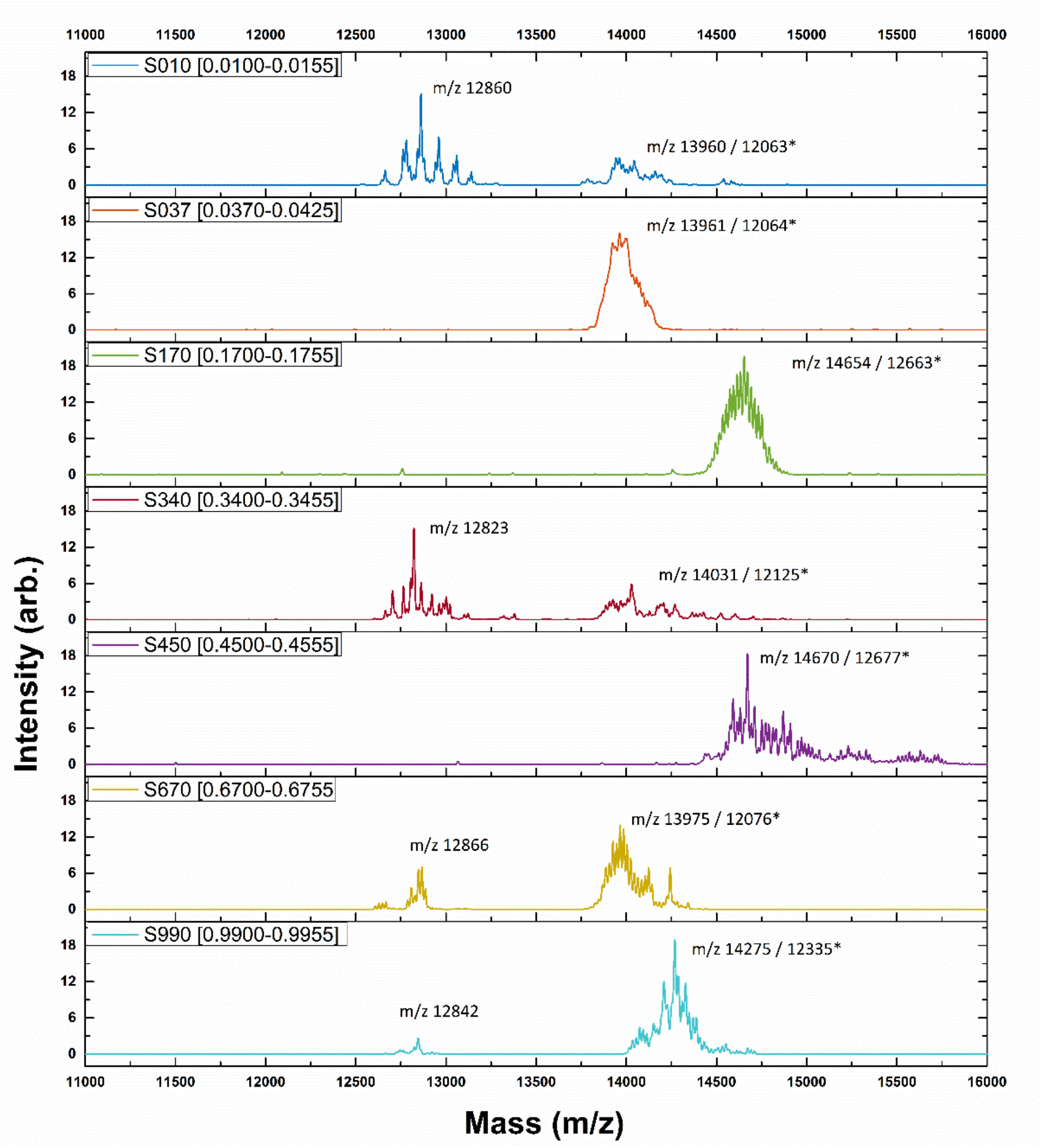

Figure S4. MS measurement used to probe resonance ejection.

Only at the correct phases can the two auxiliary HV pulses quickly trigger resonant ejection. The results demonstrate the secular characteristic of $\beta = 2/3$, with the three resonance phases sharply separated by 120°. If the phase is incorrect, no resonance occurs. However, the poor spectral line due to residual unstable ejection indicates that the modulation is also affected by the pulses. Further adjustments to their strength and polarity are needed to preserve the effectiveness of secular modulation. **Figure 9** illustrates the optimized results, where the spectral lines of the singlet and doublet in the case of $\beta = 2/3$ are identified.

Supplementary Tables

Table S1. Conversion table of ion mass and waveform frequency, given a DMZ, for β=1 the state to initiate unstable ejection.

DMZ=5			DMZ=10			DMZ=20			DMZ=40			DMZ=100			DMZ=200		
**Mass**	**frequency**	**Δ_freq**	**Mass**	**frequency**	**Δ_freq**	**Mass**	**frequency**	**Δ_freq**	**Mass**	**frequency**	**Δ_freq**	**Mass**	**frequency**	**Δ_freq**	**Mass**	**frequency**	**Δ_freq**
10000	55683.05	--	10000	55683.05	--	10000	55683.05	--	10000	55683.05	--	10000	55683.05	--	10000	55683.05	--
10005	55669.14	-13.916	10010	55655.23	-27.821	10020	55627.45	-55.600	10040	55572.02	-111.033	10100	55406.71	-276.344	10200	55134.44	-548.615
10010	55655.23	-13.905	10020	55627.45	-27.779	10040	55572.02	-55.433	10080	55461.65	-110.372	10200	55134.44	-272.270	10400	54601.72	-532.712
10015	55641.34	-13.895	10030	55599.71	-27.737	10060	55516.75	-55.268	10120	55351.93	-109.717	10300	54866.14	-268.296	10600	54084.16	-517.564
10020	55627.45	-13.884	10040	55572.02	-27.696	10080	55461.65	-55.103	10160	55242.86	-109.068	10400	54601.72	-264.417	10800	53581.04	-503.119
10025	55613.58	-13.874	10050	55544.36	-27.655	10100	55406.71	-54.940	10200	55134.44	-108.426	10500	54341.09	-260.630	11000	53091.71	-489.335
10030	55599.71	-13.864	10060	55516.75	-27.613	10120	55351.93	-54.777	10240	55026.65	-107.790	10600	54084.16	-256.933	11200	52615.54	-476.168
10035	55585.86	-13.853	10070	55489.18	-27.572	10140	55297.32	-54.615	10280	54919.49	-107.160	10700	53830.84	-253.323	11400	52151.96	-463.582
10040	55572.02	-13.843	10080	55461.65	-27.531	10160	55242.86	-54.453	10320	54812.95	-106.536	10800	53581.04	-249.796	11600	51700.42	-451.541
10045	55558.19	-13.832	10090	55434.16	-27.490	10180	55188.57	-54.293	10360	54707.03	-105.919	10900	53334.69	-246.351	11800	51260.4	-440.012
10050	55544.36	-13.822	10100	55406.71	-27.449	10200	55134.44	-54.133	10400	54601.72	-105.307	11000	53091.71	-242.984	12000	50831.44	-428.965
10055	55530.55	-13.812	10110	55379.3	-27.409	10220	55080.46	-53.974	10440	54497.02	-104.701	11100	52852.01	-239.693	12200	50413.07	-418.373
10060	55516.75	-13.802	10120	55351.93	-27.368	10240	55026.65	-53.816	10480	54392.92	-104.101	11200	52615.54	-236.476	12400	50004.86	-408.21
10065	55502.96	-13.791	10130	55324.6	-27.328	10260	54972.99	-53.658	10520	54289.41	-103.507	11300	52382.21	-233.329	12600	49606.4	-398.451
10070	55489.18	-13.781	10140	55297.32	-27.287	10280	54919.49	-53.502	10560	54186.50	-102.918	11400	52151.96	-230.253	12800	49217.33	-389.076
10075	55475.41	-13.771	10150	55270.07	-27.247	10300	54866.14	-53.346	10600	54084.16	-102.335	11500	51924.71	-227.243	13000	48837.27	-380.062
10080	55461.65	-13.760	10160	55242.86	-27.207	10320	54812.95	-53.191	10640	53982.40	-101.758	11600	51700.42	-224.298	13200	48465.88	-371.391
10085	55447.90	-13.750	10170	55215.7	-27.166	10340	54759.91	-53.036	10680	53881.22	-101.185	11700	51479	-221.416	13400	48102.83	-363.045
10090	55434.16	-13.740	10180	55188.57	-27.126	10360	54707.03	-52.883	10720	53780.60	-100.619	11800	51260.4	-218.595	13600	47747.82	-355.007
10095	55420.43	-13.730	10190	55161.48	-27.086	10380	54654.30	-52.730	10760	53680.54	-100.057	11900	51044.57	-215.834	13800	47400.56	-347.262
10100	55406.71	-13.720	10200	55134.44	-27.047	10400	54601.72	-52.578	10800	53581.04	-99.501	12000	50831.44	-213.131	14000	47060.77	-339.793
	//			//			//			//			//			//	
17980	41526.77	--	17960	41549.89	--	17920	41596.24	--	17840	41689.40	--	17600	41972.68	--	17200	42457.93	--
17985	41521.00	-5.773	17970	41538.33	-11.563	17940	41573.04	-23.193	17880	41642.74	-46.659	17700	41853.94	-118.735	17400	42213.21	-244.716
17990	41515.23	-5.770	17980	41526.77	-11.553	17960	41549.89	-23.154	17920	41596.24	-46.502	17800	41736.21	-117.733	17600	41972.68	-240.533
17995	41509.46	-5.768	17990	41515.23	-11.543	17980	41526.77	-23.115	17960	41549.89	-46.347	17900	41619.47	-116.745	17800	41736.21	-236.468
18000	41503.70	-5.766	18000	41503.7	-11.534	18000	41503.70	-23.077	18000	41503.70	-46.192	18000	41503.7	-115.771	18000	41503.7	-232.516

$$q_z = \frac{-4eV}{m{r_0}^2\Omega^2} \quad \text{(Eq. S5)}$$

Confer for the equations, (2.29) of "Quadrupole Mass Spectrometry and Its Applications", P.H. Dawson (ed.), Elsevier 1976. The conversion is based on q, the DC-parameter of Mathieu's equation. The parameter, q, is an analytic function of β, when the ion motion is a closed orbit (q = 0.908, as β = 1). Ions can be described with m (mass) and e (charges). r and z represent the radial and axial coordinate axes. Ω is the RF frequency of the alternating component of the V, the zero-to-peak amplitude potential.

Table S2. Number of steps near an integer degeneracy under different DMZs.

<table>
<tr><th>Frequency</th><th>Beta</th><th>Degeneracy</th><th colspan="6">DMZ</th></tr>
<tr><th>(Hz)</th><th>(β)</th><th>(DEG)</th><th>5</th><th>10</th><th>20</th><th>40</th><th>100</th><th>200</th></tr>
<tr><td>50086</td><td>1</td><td>2</td><td rowspan="2">6</td><td rowspan="2">3</td><td rowspan="2">2</td><td rowspan="2">1</td><td rowspan="2">1</td><td rowspan="2">1</td></tr>
<tr><td>50150</td><td>0.954</td><td>< 2.1</td></tr>
<tr><td>53880</td><td>0.666</td><td>3</td><td rowspan="2">86</td><td rowspan="2">43</td><td rowspan="2">22</td><td rowspan="2">11</td><td rowspan="2">5</td><td rowspan="2">2</td></tr>
<tr><td>54408</td><td>0.646</td><td><3.1</td></tr>
</table>

(1) Relationship between secular degeneracy, period and frequency of the in-trap motion.

$$\mathrm{DEG} = \frac{2}{\beta} = \frac{\text{period of secular oscillation}}{\text{period of micro} - \text{oscillation}} = \frac{\text{frequency of RF} - \text{waveform}}{\text{frequecy of secular oscillation}}$$

(Eq. S6)

(2) The counting of steps nearby integer degeneracy is to estimate the effectiveness of secular modulation. The smaller is the DMZ, the more effective is the modulation.
